# Interaction of WBP2 with ERα increases doxorubicin resistance of breast cancer cells by modulating *MDR1* transcription

**DOI:** 10.1038/s41416-018-0119-5

**Published:** 2018-06-25

**Authors:** Shuai Chen, Han Wang, Zhi Li, Jun You, Qiu-Wan Wu, Can Zhao, Chi-Meng Tzeng, Zhi-Ming Zhang

**Affiliations:** 1 Department of Breast Surgery, The First Affiliated Hospital of Xiamen University, 361005 Xiamen, Fujian China; 2 Translational Medicine Research Center (TMRC), School of Pharmaceutical Science, Xiamen University, 361005 Xiamen, Fujian China; 3 Key Laboratory for Cancer T-Cell Therapeutics and Clinical Translation (CTCTCT), 361005 Xiamen, Fujian China; 4 INNOVA Cell Theranostics/Clinics and TRANSLA Health Group, Yangzhou, Jiangsu China; 5 College of Pharmaceutical Sciences, Nanjing Tech University, 211816 Nanjing, China; 6 Jiansu Provincial Institute of Translation Medicine and Women-Child Health Care Hospital Affiliated to Nanjing Medical University, 210029 Nanjing, China; 7 Teaching Hospital of Fujian Medical University, 350004 Fuzhou, Fujian China

**Keywords:** Mechanisms of disease, Breast cancer

## Abstract

**Background:**

Surgery combined with new adjuvant chemotherapy is the primary treatment for early stage invasive and advanced stage breast cancer. Growing evidence indicates that patients with ERα-positive breast cancer show poor response to chemotherapeutics. However, ERα-mediated drug-resistant mechanisms remain unclear.

**Methods:**

Levels of WW domain-binding protein 2 (WBP2) and drug-resistant gene were determined by western blotting and RT-PCR, respectively. Cell viability was measured by preforming MTT assay. CD243 expression and apoptosis rate were evaluated by flow cytometry. Interactions of WBP2/ERα and ERα/MDR1 were detected by co-immunoprecipitation and chromatin immunoprecipitation (ChIP) assay, respectively.

**Results:**

There was an intrinsic link between WBP2 and ERα in drug-resistant cancer cells. Upregulation of WBP2 in MCF7 cells increased the chemoresistance to doxorubicin, while RNAi-mediated knockdown of WBP2 in MCF7/ADR cells sensitised the cancer cells to doxorubicin. Further investigation in in vitro and in vivo models demonstrated that WBP2 expression was directly correlated with MDR1, and WBP2 could directly modulate *MDR1* transcription through binding to ERα, resulting in increased chemotherapy drug resistance.

**Conclusions:**

Our finding provides a new mechanism for the chemotherapy response of ERα-positive breast tumours, and WBP2 might be a key molecule for developing new therapeutic strategies to treat chemoresistance in breast cancer patients.

## Introduction

Breast cancer is the second leading cause of cancer death among women worldwide.^1^ Chemotherapy combined with surgery is the primary treatment for patients with early stage invasive and advanced stage breast cancer.^2, 3^ Doxorubicin is commonly used in combination therapy as a fundamental drug of chemotherapy regimens.^4^ However, high proportions of patients exhibit poor initial responses to induction chemotherapy or gradually develop resistance to chemotherapy, which is perhaps the greatest obstacle for treating breast cancer. Therefore, there is significant urgency for identifying mechanisms underlying the chemotherapeutic resistance of cancer cells in order to develop treatments that are more effective for breast cancer.

ATP-binding cassette (ABC) transporters are members of a transport system superfamily that play a crucial role in the development of multidrug resistance.^5^ Numerous studies have shown that overexpression of ABC transporter genes can cause drug resistance in various cancer types.^6^ P-glycoprotein, also known as ABCB1, is encoded by *MDR1*, which belongs to the human *ABCB* (*MDR/TAP*) family and is a well-known multidrug-efflux transporter. MDR1 induces multidrug resistance utilising the energy of ATP hydrolysis to pump various chemotherapeutics drugs out of cancer cells.^7^ *MDR1* transcript levels have been indicated to be generally high in some intrinsically drug-resistant tumours, including colon cancer, renal carcinoma, hepatocellular carcinoma, pancreatic cancer and breast cancer.^8^ Moreover, MDR1 expression in breast cancer is suggestive of a more malignant phenotype.^9^ Hence, MDR1 may be a key switch molecule for the effectiveness of chemotherapeutic agents in the treatment of breast cancer.

Oestrogen receptor alpha (ERα), a nuclear receptor that is activated by the sex hormone oestrogen, is expressed in ~65% of human breast cancer.^10^ In recent years, studies have shown that patients with ERα-positive breast cancer abate the effectiveness of chemotherapeutic agents compared with patients with ERα-negative breast cancer.^11, 12^ Expression of ERα hampers paclitaxel (PTX)-induced apoptotic cell death of breast cancer cells and weakens the therapeutic efficacy of PTX in vivo.^13, 14^ Besides, ERα has been verified to contribute to drug resistance of breast cancer via activation of DNA methyltransferases and regulating the expression of ABC transporters.^15, 16^ For instance, ERα-positive drug-resistant MCF7/PTX cells show higher global DNA methylation than ERα-negative drug-resistant MDA-MB-231/PTX cells.^17^ In addition, ERα can directly activate *MDR1* transcription in ERα-positive breast cancer cells via binding to the *MDR1* promoter with the help of SP1, suggesting that ERα may be critical to developing novel therapeutic strategies for overcoming drug resistance of breast cancer cells in the future.^15^ Nonetheless, while studies have illustrated that ERα contributes to the promotion of cell proliferation, of cell apoptosis, and regulation of intracellular drug concentration in some drug resistance cells, additional underlying mechanisms for ERα-mediated drug resistance, including potential technologies and strategies for improving chemotherapeutic sensitivity require further probing.^18, 19^

WW domain-binding protein 2, encoded by the *WBP2* gene, is a breast cancer oncogene.^20, 21^ WBP2 serves as a molecular on/off switch that controls the crosstalk between ERα,^22^ WWOX,^23^ Wnt^24^ and Hippo signalling networks.^25^ As a co-activator of ER, WBP2 binds to ERα directly and activates proliferation-related target genes expression to promote the pathogenesis and progression of breast cancer.^24^ As described above, ERα is critical for chemotherapy resistance in breast cancer. However, there is no evidence that shows that the interaction between WBP2 and ERα contributes to drug resistance in ERα-positive drug-resistant breast cancer cells during chemotherapy.

Herein, we determined the differential expression of WBP2 in MCF7 cells and drug-resistant MCF-7/ADR cells. The in vitro data illustrated that WBP2 suppressed doxorubicin-induced cell death and reduced the sensitivity of chemotherapy agents. Next, we explored the underlying mechanism of WBP2-mediated drug resistance. We found that WBP2 could upregulate MDR1 expression in MCF7 cells, and ERα was required for WBP2-mediated *MDR1* transcriptional activation. In an in vivo experiment, we further confirmed the role of WBP2 on the sensitivity of chemotherapy drugs. Together, our data demonstrate that WBP2 may decrease the sensitivity of doxorubicin to drug-resistant MCF-7/ADR cells by promoting *MDR1* transcription through interaction with ERα.

## Materials and methods

### Cell culture, transfection and cell line construction

MCF-7, BT474 and MDA-MB-231 cell lines were purchased from American Type Culture Collection (ATCC; Manassas, VA) and MCF-7/ADR, MCF-7/DDP and MDA-MB-231/ADR cell lines were from KeyGen Biotech. Inc (NanJing, China). All cells were cultured according to the manufacturer’s instructions. MCF-7/ADR and MDA-MB-231/ADR cell lines were maintained in RPMI-1640 medium supplemented with 10% foetal bovine serum (FBS) and 1 μM doxorubicin. MCF-7/DDP cells were grown in DMEM medium with 1 μM cisplatin. RNAi-mediated knockdown of WBP2 was performed using WBP2 siRNA (GAACUCACAUUCAAUGACA). For the construction of WBP2 stable expression cell line, MCF-7/MDA-MB-231 cells transfected with EGFP and EGFP-WBP2 plasmids for 24 h were screened by G418. Transfection of WBP2 siRNA into MCF-7/ADR cells was performed when cells were 30–50% confluent.

### Cell viability assay

Cells were seeded in the 96-well plates at a density of 3 × 10^3^ cells/well. For inhibitor (fulvestrant) assay, cells were pre-treated with 100 nM fulvestrant (HY-13636, MCE, USA) for 48 h before seeding. After incubation for 24 h, cells were treated with 0, 0.2, 0.5 and 1 μM doxorubicin for 24, 48 and 72 h. At each time point, MTT reagents (Sigma-Aldrich, USA) were added to cells and were maintained in the dark at 37 °C for another 4 h. Then, cells stained with formazan were dissolved with 150 μL DMSO (Sigma-Aldrich, USA) after removing the medium. OD values were measured at the wavelength of 492 nm using an ELISA plate reader.

### Flow cytometry

For apoptosis assay, cells were seeded in the 6-well plates and were treated with doxorubicin when cells were 70–80% confluent. In WBP2 stable expression MCF-7 and control groups, 0.2 and 0.5 μM doxorubicin were added to cells for 24 h. MDA-MB-231 cells transfected with WBP2 siRNA were treated with 0.5 and 1 μM doxorubicin for 72 h. Then, cells were washed twice with ice-cold PBS buffer. After adding 400 μL binding buffer to cells, 5 μL Annexin V-APC (KeyGen Biotech. Inc, NanJing, China) were incubated with cells for 15 min in the dark at room temperature. Next, 5 μL PI were added to cells and the proportion of apoptotic cells were analysed by flow cytometry.

For the detection of cell surface-bound CD243 (*ABCB1* or *MDR1*, 17-0441-82, eBioscience, USA), we performed indirect fluorescence staining and flow cytometry analysis according to the manufacturer’s protocol. Cells were washed with ice-cold PBS containing 1% FBS and 0.09% NaN_3_. After centrifugation, cells were then resuspended in PBS containing 1% FBS and 0.09% NaN_3_ and 50 μL cell suspension (~1 × 10^6^ cells) were removed for incubation with IgG2a K Isotype Control APC and CD243 (ABCB1) APC antibodies (Abcam, UK) for 20 min on ice. After washing twice with PBS buffer and transient centrifugation, cells were suspended again, and then analysed with flow cytometry.

### In vivo drug sensitivity assay

Female BALB/c nude mice (5–6 weeks of age, 16–18 g) were obtained from the Laboratory Animal Center of Xiamen University, China. Mice were housed in standard conditions with free access to food and water. All experimental procedures were approved by The Animal Welfare Committee of Research Organization (X201011), Xiamen University.

All mice were randomly divided into four groups, and a xenograft model was established by subcutaneous injection of 0.2 mL (2 × 10^6^) MCF7-EGFP control cells and WBP2 stable expression cells. Once tumour volume grew to ~1 cm^3^, four groups of MCF7-bearing mice (*n* = 7–8 per group) were, respectively, treated with PBS (0.1 mL, tail i.v. injection), adriamycin (0.1 mL, 10 mg/kg, tail i.v. injection) and housed for another 12 consecutive days. Tumour size and mice body weights were measured on days 10, 12, 14, 16, 18, 20, 22, 24 and 26 after initial injection. Tumour volume was evaluated using the formula: Volume = 0.5 × a × b^2^, where a and b indicated the length and width of tumour, respectively. All mice were killed by cervical dislocation and the tumours were harvested. After weighing, all tumours were stored at −80 °C until analysis.

### Western blotting and co-immunoprecipitation

Western blotting was in accordance with the previous instruction.^26^ Protein samples were separated with 8–12% SDS-PAGE and protein levels were determined with primary antibodies against WBP2 (GeneTex, USA), caspase-3, PARP, cleaved PARP (Cell Signaling Technology, USA), MRP1 (Sangon Biotech, China), MDR1 (Abcam, UK) and BCRP (Sangon Biotech, China). For co-immunoprecipitation experiments, cell lysates were incubated with WBP2 and ERα (Santa Cruz Biotechnology, USA) antibodies overnight and the complexes were incubated with protein A/G beads for another 3 h at RT. Then the compounds resuspended in 2× SDS loading buffer were boiled for 5 min and then the interaction between WBP2 and ERα were analysed by western blotting.

### Real-time qPCR

Total RNA was extracted using TRIzol Reagent (Roche, USA) according to the manufacturer’s protocol. After performing reverse transcriptional PCR reaction using First-Strand cDNA Synthesis Kit, relative mRNA levels were evaluated by qPCR reaction using SYBR Green (Roche, Indianapolis, IN, USA) with specific primers (Table 1). GAPDH served as the internal control. Relative gene expression was calculated using the 2^−∆∆Ct^ method.

**Table 1:** Primers for quantitative real-time PCR

Gene	Forward sequence (5′–3′)	Reverse sequence (5′–3′)
*WBP2*	GCGGAGTGATCGTCAATAACA	GACCCGGTAAGGGGTAAGGT
*MDR1*	TCGTTTCCTTTAGGTCTTTCCAC	CTTCTTCTTTGCTCCTCCATTGC
*BCRP*	CCACAGGTGGAGCAAATCT	TCGCGGTGCTCCATTTATCA
*MRP1*	CGCTCTGGGACTGGAATGT	ACCCACACTGAGGTTGGTTA
*GAPDH*	GGAGCGAGATCCCTCCAAAAT	GGCTGTTGTCATACTTCTCATGG

### Luciferase reporter assay

For the construction of reporter plasmid, the MDR1 promoter region was cloned into the pGL6 reporter plasmid (318 bp, forward primer: 5′-CAGGGTACCAGTTGAAATGTCCCCAATGAT-3′, reverse primer: 5′-CCTAGATCTGGAAAGACCTAAAGGAAACGAAC-3′). Briefly, MCF-7 cells transfected with pGL6-MDR1 reporter plasmid were seeded into 24-well plates and incubated for another 24 h. Then, cells were digested with 100 μL ice-cold lysis buffer after washing for twice with PBS buffer. Luciferase reporter activity was measured with a Victor 3V multi-label plate reader (Perkin Elmer).

### Chromatin immunoprecipitation (ChIP) assay

CHIP assay was processed according to the manufacturer’s instruction utilising CHIP assay kit (17–371, Merck-Millipore, USA). The primers for qCHIP on MDR1 were GCGTTTCTCTACTTGCCCTTTC (forward) and AGCCAATCAGCCTCACCACAG (reverse). Calculation of standard curves was in accordance with the previous experimental method.^27^ The signal differences were calculated using the 2^−∆∆Ct^ method.

### Immunohistochemistry

Human breast cancer tissue was obtained from the First Affiliated Hospital of Xiamen University. IHC experiment was performed according to a previously published standard protocol.^28^ Primary antibodies against WBP2 (GeneTex, USA) and MDR1 (Abcam, UK) were used to evaluated the expression levels of WBP2 and MDR1 in breast cancer tissues.

### RNA sequencing

RNA was extracted from control (EGFP) and WBP2 stable expressed MCF7 cells (EGFP-WBP2) using TRIzol Reagent. Then RNA sequencing was performed by Aksomics Inc (Shanghai, China). Results of statistical analysis were based on GEO databases, GO and KEGG enrichment analysis.

### Data mining

GOBO Gene Set Analysis was used to evaluate the association between WBP2 expression and overall survival in ER-positive cancer patients (*n* = 560; Chin, GSE1456, GSE3494 and GSE7390 studies). Breast Cancer Gene Expression Miner v4.1 was used to explore the correlation between WBP2 and ER (*ESR1*) expression in ER-positive breast cancer patients (*n* = 3762). The correlation between MDR1 and WBP2 in breast cancer patients (*n* = 1090) was analysed utilising TCGA data set and SPSS software. Correlational analysis between WBP2 and MDR1 in chemotherapy treatment patients (*n* = 114) was performed using Oncomine database.

### Statistical analysis

All experiments were repeated for three independent times. Data were presented as the mean ± SEM. Correlation analysis was processed by using SPSS software. Data analysis was analysed by one-way analysis of variance (ANOVA) or Student’s unpaired *t* test using GraphPad Software, Prism 5.0. *P* < 0.05 was defined as the statistically significant differences.

## Results

### WBP2 is overexpressed in doxorubicin-resistant ERα-positive breast cancer cells

**Fig. 1:**
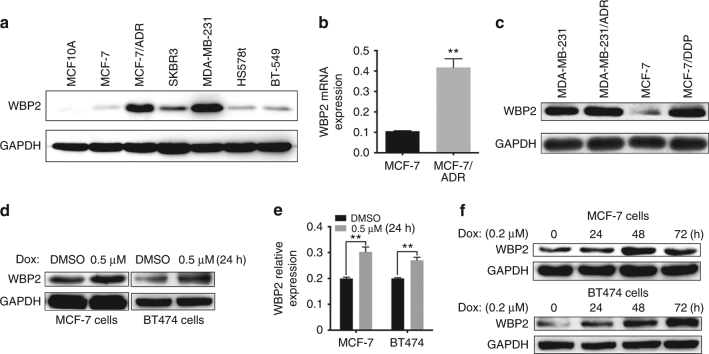
Correlation between WBP2 and doxorubicin resistance in ER-positive breast cancer cells. **a** Protein levels of WBP2 in normal breast epithelial cells (MCF10A) and breast cancer cells (MCF-7, MCF-7/ADR, SKBR3, MDA-MB-231, HS578t and BT-549). **b** Relative expression of *WBP2* mRNA in MCF-7 and MCF-7/ADR cells. **c** Protein levels of WBP2 in ER-negative breast cancer cells (MDA-MB-231 and MDA-MB-231/ADR) and ER-positive breast cancer cells (MCF-7 and MCF-7/DDP). Differential protein expression (**d**) and mRNA expression (**e**) of WBP2 in MCF-7 and BT474 cells treated with DMSO or 0.5 μM doxorubicin for 24 h. **f** Differential protein expression of WBP2 in MCF-7 and BT474 cells treated with 0.2 μM doxorubicin for 0, 24, 48 and 72 h. Dox indicates doxorubicin. **P < 0.01

Various studies have indicated that WBP2 is involved in the development of breast cancer by affecting multiple signal mechanisms.^22, 25, 29^ However, the expression pattern of WBP2 in breast cancer has not been investigated. Herein, we evaluated the expression levels of WBP2 in human normal breast epithelial cells MCF10A and several types of breast cancer cells. We found a higher expression of WBP2 in breast cells than in normal breast epithelial cells, especially in highly aggressive breast cancer cells (MDA-MB-231 and SKBR3) or doxorubicin-resistant MCF7/ADR cells (Fig. 1a). Importantly, we observed that WBP2 mRNA and protein levels were significantly increased in MCF7/ADR compared with MCF7 cells (Fig. 1a, b). To ascertain the doxorubicin resistance of MCF7/ADR cells, we evaluated cell viability in both MCF7 and MCF7/ADR cells under varying doses of doxorubicin. We observed a strong doxorubicin resistance in MCF7/ADR cells in contrast to MCF7 cells (Fig. S1A-B). WBP2 was also highly expressed in MCF7/DDP cells compared with its parental doxorubicin-sensitive control cells, but no difference in WBP2 expression was observed between ERα-negative MDA-MB-231/ADR and MDA-MB-231 cells (Fig. 1c), implying WBP2 upregulation in doxorubicin-resistant cells was related to ER status. We also evaluated WBP2 level in breast cancer patients and found WBP2 was moderately upregulated in ER-positive (*n* = 11) compared with ER-negative breast cancer patients (*n* = 4) (Fig. S1C-D). This differential expression was further confirmed through data analysis using Breast Cancer Gene Expression Miner v4.1 (Fig. S1E). Thus, we next determined the relationship of WBP2 and doxorubicin resistance in ER-positive breast cancer cells. Upon treatment with doxorubicin, WBP2 transcription and protein levels were upregulated in MCF-7 and BT474 cells which was also ER positive (Fig. 1d, e). A time-dependent increase in WBP2 expression was also observed until 48 h in MCF-7 and 24 h in BT474 cells after treatment with 0.2 μM doxorubicin (Fig. 1f). These findings suggest that WBP2 is upregulated in doxorubicin-resistant MCF-7/ADR cells, indicating its potential role in facilitating doxorubicin resistance in breast cancer.

### WBP2 affects the doxorubicin resistance phenotype of breast cancer cells

**Fig. 2:**
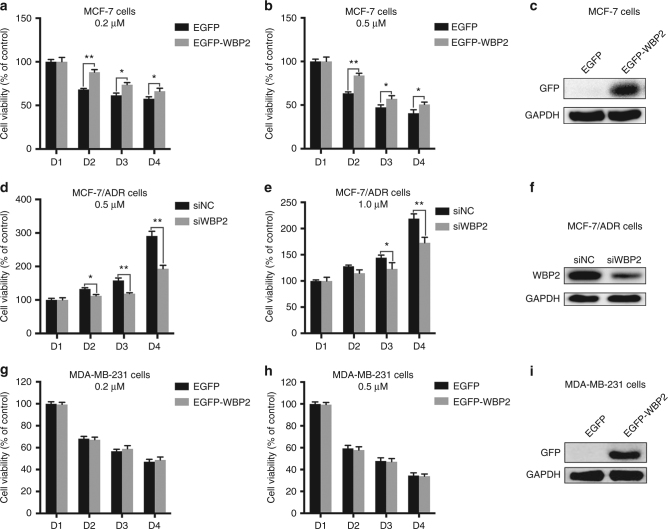
Effects of WBP2 on cell viability in doxorubicin-treated MCF-7 and MCF-7/ADR cell viability. Measurement of cell viability using MTT assay in control MCF-7 cells and WBP2-overexpressing MCF-7 cells treated with 0.2 μM (**a**) and 0.5 μM (**b**) doxorubicin for 0, 24, 48 and 72 h. **c** WBP2 overexpression efficiency was verified by performing western blotting in MCF-7 cells. Measurement of cell viability using MTT assay in MCF-7/ADR cells and RNAi-mediated WBP2 knockdown in MCF-7/ADR cells treated with 0.5 μM (**d**) and 1.0 μM (**e**) doxorubicin for 0, 24, 48 and 72 h. **f** Validation of WBP2 knockdown efficiency in MCF-7/ADR cells. Test of cell viability through MTT assay in control MDA-MB-231 cells and WBP2-overexpressing MDA-MB-231 cells treated with 0.2 μM (**g**) and 0.5 μM (**h**) doxorubicin for 0, 24, 48 and 72 h. **i** Validation of WBP2 overexpression efficiency in MDA-MB-231 cells. EGFP, control MCF-7 cells; EGFP-WBP2, WBP2-overexpressing MCF-7 cells; siNC, control MCF-7/ADR cells; siWBP2, RNAi-mediated WBP2 knockdown in MCF-7/ADR cells. *P < 0.05, **P < 0.01

To explore the effect of WBP2 on the sensitivity of doxorubicin in MCF-7 cells, we generated a WBP2-stably expressed cell line in MCF-7 cells. First, we investigated whether exogenous WBP2 affected the cell viability of MCF-7 cells in the presence of doxorubicin. As shown in Fig. 2a, upon exposure to 0.2 μM doxorubicin, cell viability showed a rapid decrease in MCF-7 cells from days 2 to 4, but the rate of cell viability had a significant improvement in WBP2 overexpressed MCF-7 cells. In comparison to MCF-7 cells, doxorubicin sensitivity decreased in WBP2 overexpressed cells when treated with 0.5 μM doxorubicin (Fig. 2b). WBP2 overexpression efficiency was shown in the right panel (Fig. 2c). To determine whether the decrease of drug sensitivity was due to exogenous WBP2-mediated overproliferation, we performed MTT assay in WBP2 overexpressed/silenced MCF-7 and BT474 cells and found cell growth ability was moderately increased in WBP2 overexpressed MCF-7 cells and moderately decreased in WBP2-silenced MCF-7 cells from D3 to D4, but no significant differences were observed in comparison to control group cells (Fig. S2A). In BT474 cells, both WBP2 upregulation or depletion have no influence on cell growth (Fig. S2B), suggesting that WBP2 upregulation did not affect cell growth in ER-positive cells.

Conversely, the knockdown of WBP2 by RNAi robustly reduced the doxorubicin-resistance phenotype of MCF-7/ADR cells. Cell viability of MCF-7/ADR was influenced in the presence of doxorubicin, but ablation of WBP2 enhanced the doxorubicin sensitivity of MCF-7/ADR cells and significantly inhibited the cell survival rate of MCF-7/ADR in 0.5 μM and 1 μM doxorubicin (Fig. 2d, e). Moreover, we determined the role of WBP2 in the sensitivity of doxorubicin in ERα-negative MDA-MB-231 cells. Unlike MCF-7 cells, there was no significant difference in cell viability between control MDA-MB-231 cells and WBP2-transfected MDA-MB-231 cells after treatment with 0.2 μM and 0.5 μM doxorubicin (Fig. 2g, h). WBP2 knockdown efficiency in MCF-7/ADR and MDA-MB-231 cells were validated by western blotting (Fig. 2f, i). Thus, our results illustrate the impact of WBP2 on cell viability following doxorubicin treatment in ERα-positive/negative breast cancer cells.

### Effects of WBP2 on doxorubicin-induced cell apoptosis in ERα-positive breast carcinoma

**Fig. 3:**
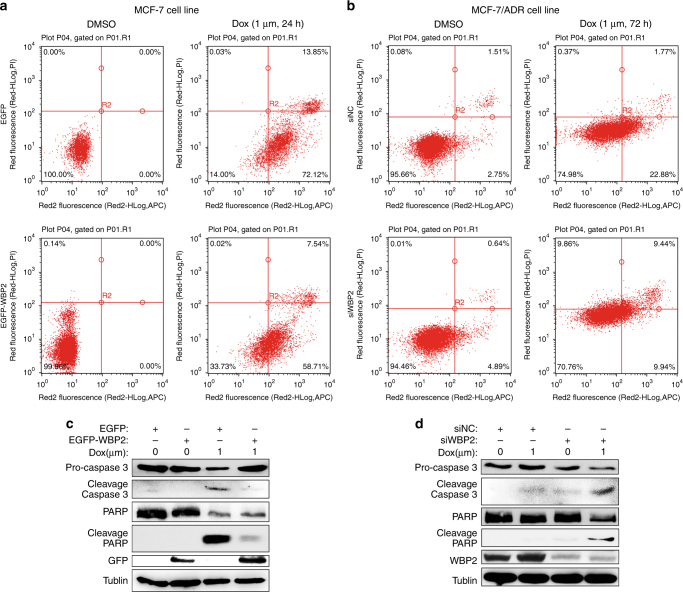
Role of WBP2 in doxorubicin-induced cell apoptosis in MCF-7 and MCF-7/ADR cells. **a** Cell apoptosis analysis utilising flow cytometry in control MCF-7 cells and WBP2-overexpressing MCF-7 cells treated with 1.0 μM doxorubicin for 24 h. **b** Detection of cell apoptosis utilising flow cytometry in MCF-7/ADR cells and RNAi-mediated WBP2 knockdown in MCF-7/ADR cells treated with 1.0 μM doxorubicin for 72 h. **c** Cell lysates from the control MCF-7, control MCF-7-WBP2, drug-treated MCF-7 and drug-treated MCF-7-WBP2 cells were separated by 8–12% SDS-PAGE, blotted and probed with antibodies against caspase-3, PARP, cleaved PARP, GFP and WBP2. Tubulin served as the internal control. **d** The expression of above four antibodies (caspase-3, PARP, cleaved PARP and WBP2) were also detected in the control MCF-7/ADR, control MCF-7/ADR-siWBP2, drug-treated MCF-7/ADR and drug-treated MCF-7/ADR-siWBP2 cells by western blotting. Tubulin served as the internal control. EGFP, control MCF-7 cells; EGFP-WBP2, WBP2 stable expression MCF-7 cells; siNC, control MCF-7/ADR cells; siWBP2, RNAi-mediated WBP2 knockdown in MCF-7/ADR cells

We also investigated whether WBP2 contributed to the development of resistance to doxorubicin-induced cell apoptosis in MCF-7 cells. We observed that doxorubicin markedly induced cell apoptosis in MCF-7 cells (Fig. 3a, upper panels), but forced expression of WBP2 in MCF-7 suppressed the proportion of apoptotic cells evoked by doxorubicin (Fig. 3a, lower panels). As observed in Fig. 3b (upper panels), doxorubicin moderately induced cell apoptosis in MCF-7/ADR cells transfected with negative control siRNA, but the rate of apoptotic cells was significantly less than doxorubicin-treated MCF-7 cells (25.02% vs. 86%). However, WBP2 siRNA-transfected MCF-7/ADR cells showed a decrease in doxorubicin-resistance phenotype. We found that treatment with doxorubicin promoted the proportion of late apoptosis and necrosis in WBP2 siRNA-transfected MCF-7/ADR cells compared with MCF-7/ADR cells transfected with control siRNA (Fig. 3b, lower panels, 19.3% vs. 2.14%). However, there was a decline of proportion of early apoptosis cells in MCF-7/ADR-siWBP2 cells compared with MCF-7/ADR-siNC cells (9.94% vs. 22.88%).

To confirm the critical role of WBP2 in regulating doxorubicin-induced cell apoptosis, we evaluated the expression of apoptosis-related proteins in MCF-7 and MCF-7/ADR cells in the presence of doxorubicin. For this purpose, MCF-7 and MCF-7 cells transfected with WBP2 were treated with 1 μM doxorubicin for 24 h. We observed that doxorubicin dramatically activated the cleavage of caspase-3 and PARP in MCF-7 cells (Fig. 3c, the third column), but overexpression of WBP2 in MCF-7 cells significantly inhibited doxorubicin-induced activation of caspase-3 and PARP (Fig. 3c, the fourth column). On the contrary, knockdown of WBP2 enhanced doxorubicin-induced cleavage of caspase-3 and PARP in MCF-7/ADR in comparison with the control MCF-7/ADR cells, which had no response to doxorubicin treatment (Fig. 3d, the third row vs. the fourth row). These data indicate that WBP2 affects doxorubicin-induced cell apoptosis in breast cancer.

### WBP2 is involved in the doxorubicin resistance of breast carcinoma in vivo

**Fig. 4:**
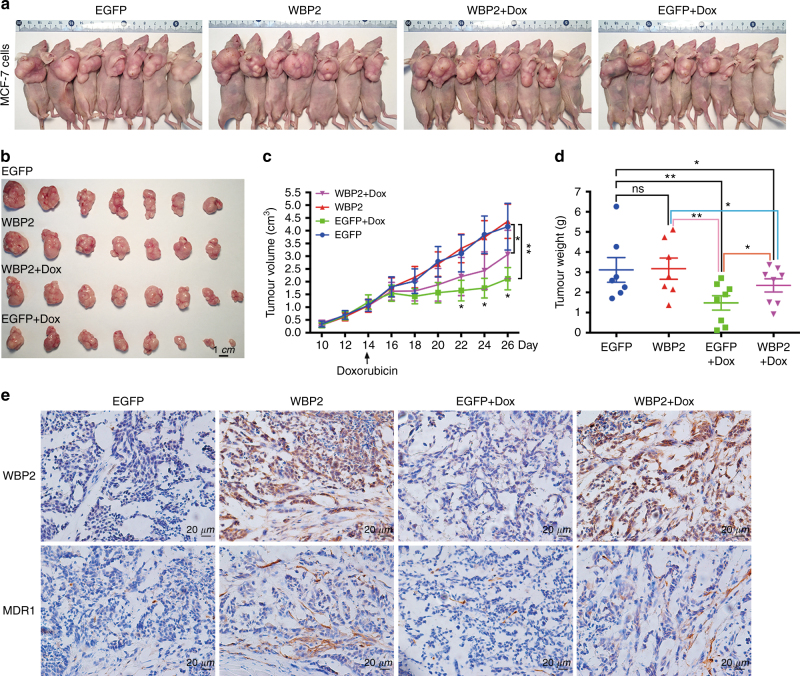
Drug resistance of WBP2 to doxorubicin in nude mice bearing breast cancer tumours. **a** All mice were killed by cervical dislocation and imaged. **b** The tumours of mice from the four groups were harvested and imaged. **c** The tumour growth curve of xenograft mice after initial cell injection and drug treatment. **d** The differences of tumour weight among the four groups. **e** IHC staining of tumours with WBP2 and MDR1 antibodies in the four groups. Scale bar, 1 cm. ns no significant. *P < 0.05, **P < 0.01

Next, the antitumour effects of doxorubicin in tumour-bearing nude mice were examined. A mouse tumour model was generated by endermic injection of control MCF-7 cells and WBP2 overexpressed MCF-7 cells. Once the tumour volume grew to ~1 cm^3^, nude mice bearing tumours were treated with PBS or doxorubicin. Compared with PBS-treated mice, tumours from both the control group and the WBP2 overexpression group were significantly suppressed after doxorubicin treatment. However, the tumours of the WBP2 overexpression group showed weaker sensitivity to doxorubicin administration than the tumours of the control MCF-7 cell group. As shown in Fig. 4b, the tumour sizes of WBP2 overexpression group treated with doxorubicin were larger than control group mice. Furthermore, the tumour growth curve visually showed that mice injected with WBP2-overexpressing MCF-7 cells facilitated the development of resistance to doxorubicin in comparison with the control group mice (Fig. 4c). The tumour weight loss caused by drug treatment was lower due to WBP2 overexpression, compared to the doxorubicin-treated control mice (Fig. 4d). We further explored whether mice body weight would affect the tumour size of doxorubicin-treated controls and WBP2 overexpression mice. We observed a significant body weight loss after drug injection compared with PBS injection. However, no body weight differences were detected between doxorubicin-treated control and WBP2 overexpression mice (Fig. S3). This indicated that WBP2-mediated doxorubicin resistance of breast carcinoma in vivo was independent of body weight changes. Expression of multidrug resistance proteins is the main cause of drug resistance. Therefore, we performed immunohistochemical staining using WBP2 and MDR1 antibodies to determine MDR1 expression in nude mice bearing breast cancer tumours. Consistent with the in vitro results, there was higher MDR1 expression in WBP2 overexpression group treated with/without doxorubicin (Fig. 4e, second and fourth bottom columns), indicating that the drug resistance of WBP2 overexpression group was due to the upregulation of MDR1 expression. Thus, our findings confirm that WBP2 upregulation also enhanced the resistance effect of doxorubicin in nude mice bearing breast cancer tumours.

### WBP2 is correlated with the expression of MDR1 in breast cancer

**Fig. 5:**
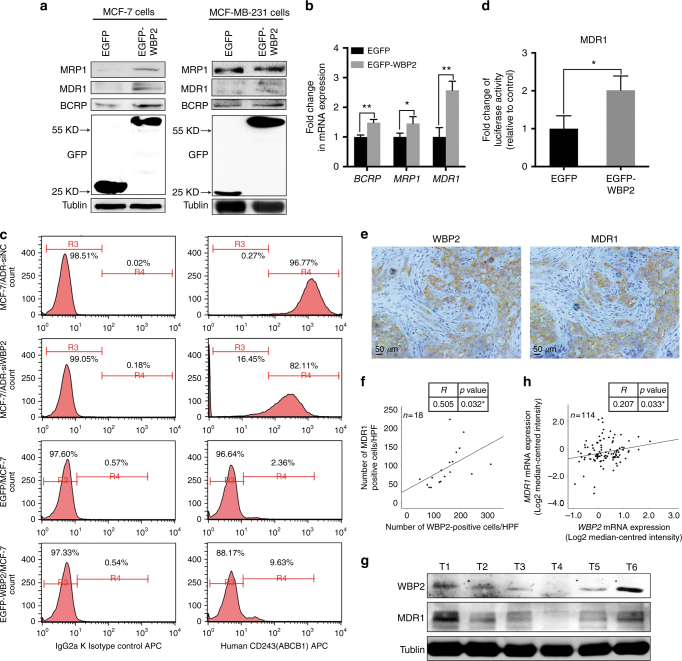
Effects of WBP2 on the expression of MDR1 in ER-positive MCF-7 cells. **a** Cell lysates from control MCF-7 and WBP2-overexpressing MCF-7 cells were separated by 10% SDS-PAGE, blotted and probed with antibodies against MRP1, MDR1, BCRP and GFP. Tubulin served as the internal control. **b** Transcription levels of *MRP1*, *MDR1* and *BCRP* were tested by using real-time quantitative PCR. *GAPDH* served as the internal control. **c** Detection of MDR1 expression in MCF-7/ADR-siNC, MCF-7/ADR-siWBP2, control MCF-7 and WBP2-overexpressing MCF-7 cells by indirect fluorescence staining and flow cytometry analysis. **d** Luciferase activity of MDR1-reporter gene in control MCF-7 and WBP2 stable expression MCF-7 cells. **e** IHC staining with WBP2 and MDR1 antibodies in clinical breast cancer specimens. **f** Correlation analysis between the number of WBP2-positive cells and MDR1-expressed cells. Averages of five HPFs per sample was used to evaluate the Pearson correlation (*n* = 18). R indicates Pearson’s coefficient. **g** The protein levels of WBP2 and MDR1 in clinical breast cancer specimens were detected by western blotting. **h** Correlation analysis between WBP2 and MDR1 expression in chemotherapy treatment patients (*n* = 114). R indicates Pearson’s coefficient. Tubulin served as the internal control. EGFP, control MCF-7 cells; EGFP-WBP2, WBP2-overexpressing MCF-7 cells. Scale bar, 50 μm. *P < 0.05, **P < 0.01

As aforementioned, expression of multidrug resistance-associated proteins is the primary reason of failure in chemotherapy. Since WBP2 participated in the process of doxorubicin resistance of breast cancer, we assessed the expression of several MDR molecules including MRP1, MDR1 and BCRP in MCF-7 and MCF-7 cells transfected with the WBP2 plasmid. As shown in Fig. 5a, b, MCF-7 cells treated with exogenous WBP2 (EGFP-WBP2 group) significantly enhanced the protein and mRNA levels of *MRP1*, *MDR1* and *BCRP* compared with control MCF-7 cells (EGFP group). Nevertheless, we did not find any changes of MRP1, MDR1 and BCRP protein levels in MDA-MB-231 cells treated with exogenous WBP2 (Fig. 5a, right images). In consideration of the most prominent change of MDR1 and its close connection with ERα, we considered MDR1 as the potential targeted regulator of WBP2. Hence, expression of MDR1 (ABCB1) was also examined by indirect fluorescence staining and flow cytometry analysis in MCF-7 and MCF-7/ADR cells. We found that 96.77% of MCF-7/ADR cells expressed MDR1, but the positive peak vividly shifted leftward and showed the decrease of cells expressed MDR1 (82.11%) in the absence of WBP2 in MCF-7/ADR cells (Fig. 5c, the first right row vs. the second right row). However, there was hardly any MDR1 protein expression in MCF-7 cells. However, upregulation of WBP2 in MCF-7 cells notably promoted the number of MDR1-positive cells (Fig. 5c, the fourth right row vs. the third right row). Although MDR1 expression had increased nearly fourfold in WBP2 overexpressed cells, MDR1-positive cells were only 10% of cells. In our view, the cause of this issue was likely that MDR1 positioned on the cell membrane usually was a lagging indicator after its overproduction induced by WBP2 upregulation in the cells. Next, we constructed a PGL3 reporter plasmid containing the MDR1 promoter fused to a firefly luciferase reporter gene to verify whether WBP2 directly regulated MDR1 expression. A luciferase assay showed that overexpression of WBP2 augmented *MDR1* reporter gene activity in MCF-7 cells, indicating that MDR1 expression was modulated by WBP2 in breast cancer cells (Fig. 5d).

To further explore the relationship between WBP2 and MDR1, we subsequently performed IHC staining of WBP2 and MDR1 in 18 cases of human fresh breast cancer specimens. Staining results of serial sections of 18 samples showed that most cells of WBP2-positive staining had MDR1 expression (Fig. 5e). The correlation analysis using image pro plus and SPSS software showed that the number of WBP2-positive cells was positively associated with MDR1-expressed cells in these breast cancer samples (Fig. 5f). Furthermore, western blotting was conducted to determine the protein levels of WBP2 and MDR1 in human breast cancer tissues. Similarly, breast cancer tissue with high expression of WBP2 showed higher expression of MDR1 than breast samples with low WBP2 expression (Fig. 5g).

To obtain more supporting evidence to validate our findings, we analysed the correlation between MDR1 and WBP2 using TCGA data set, which contained 1090 breast cancer patients and result showed that there was a slight positive relativity between MDR1 (*ABCB1*) and WBP2 (*p* = 0.050) (Fig. S4A). However, when we performed association analysis of MDR1 and WBP2 in chemotherapy treatment patients by using the publicly available Oncomine database (Perou Breast, Sorlie Breast, and Sorlie Breast 2 Statistics), the analysis result revealed a considerable positive correlation between MDR1 and WBP2 in 114 chemotherapy treatment patients (Fig. 5h). Notably, we also discovered that WBP2 expression correlated positively with overall survival in 560 ER-positive cancer patients (Chin, GSE1456, GSE3494 and GSE7390 studies) by using GOBO Gene Set Analysis, which suggested that WBP2 was associated with progression and poor clinical outcomes of ER-positive tumours (Fig. S4B). Besides, WBP2 was positively correlated with ER (*ESR1*) expression in 3762 ER+ breast cancer patients utilising Breast Cancer Gene Expression Miner v4.1 (Fig. S4C). Therefore, our results indicate that WBP2 expression is highly correlated with MDR1 expression in chemotherapy treatment breast cancer patients, and WBP2, ER and MDR1 gene signature might be served as a predictive marker for resistant to chemotherapy in breast cancer patients.

### The binding of ERα to WBP2 promotes the activity of ERα in modulating *MDR1* transcription

**Fig. 6:**
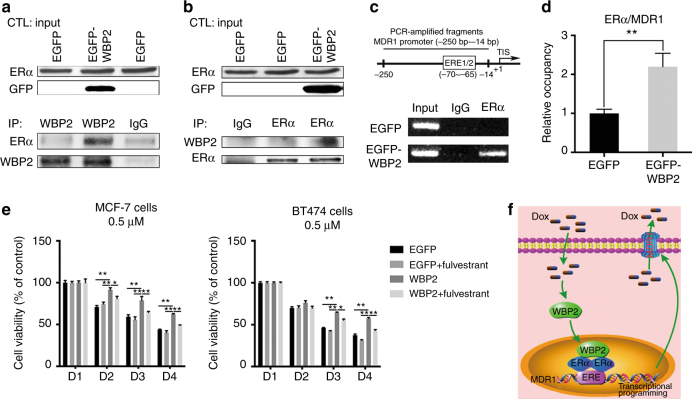
WBP2 mediates the upregulation of MDR1 in MCF-7 cells. The interaction between WBP2 and ERα were detected using antibodies against WBP2 (**a**) and ERα (**b**) by performing co-immunoprecipitation assay in control MCF-7 and WBP2-overexpressing MCF-7 cells. **c** ChIP assay was performed to determine the interaction between the half-ERE motif of MDR1 promoter region and ERα using anti-ERα antibody in control MCF-7 and WBP2 stable expression MCF-7 cells. **d** The association of ERα with the MDR1 promoter was confirmed by qChIP assay. **e** After pre-treated with fulvestrant for 48 h, cell viability was measured using MTT assay in control MCF-7/BT474 cells and WBP2-overexpressing MCF-7/BT474 cells under the treatment with 0.5 μM doxorubicin for 0, 24, 48 and 72 h. **f** The proposed model of WBP2-mediated drug resistance in ER-positive breast cancer cells. EGFP, control MCF-7 cells; EGFP-WBP2, WBP2-overexpressing MCF-7 cells. *P < 0.05, **P < 0.01

Previous studies confirmed that WBP2 is a co-activator of ERα and WBP2 is conducive to the transcriptional activity of ERα through binding to ERα.^24^ Moreover, a previous report verified that ERα directly activated *MDR1* transcription in PTX-induced resistance of ERα-positive breast cancer cells.^15^ Thus, we hypothesised that ERα was the key determinant that affected the expression pattern between WBP2 and MDR1 in breast cancer. To test this hypothesis, a co-immunoprecipitation assay was first performed using antibodies against WBP2 and ERα in MCF-7 cells (EGFP control group and EGFP-WBP2 group). Our results showed that WBP2 upregulation robustly enhanced the binding of ERα to WBP2, indicating that WBP2 overexpression possibly recruited more ERα protein to promote the doxorubicin resistance of MCF-7 cells (Fig. 6a, b). To investigate the mechanism further, we amplified the promoter region of human MDR1 as a target for ChIP assays (from −250 to −14 bp relative to the translational start site that contained the atypical oestrogen response element). The ChIP assay was processed to determine the interaction of MDR1 and ERα utilising the specific antibody against ERα. The presented data revealed that the atypical oestrogen response element of MDR1 was specifically immunoprecipitated using ERα antibody in MCF-7 cells treated with exogenous WBP2, but the immunoprecipitation of ERα and MDR1 was hardly detected in control MCF-7 cells (Fig. 6c). The quantitative ChIP (qChIP) assay also verified an association of ERα with the MDR1 promoter region in WBP2-overexpressing MCF-7 cells than control MCF-7 cells (Fig. 6d).

Since WBP2 upregulation mediated the increase of MDR1 and chemoresistance was generated via recruiting ERα, we thought blockage of ER potentially contributed to overcome WBP2 overexpression evoked resistance to chemotherapy. Therefore, we detected the cytotoxicity of doxorubicin on WBP2 overexpressed MCF-7 and BT474 cells with or without fulvestrant treatment. As observed, WBP2 overexpression significantly enhanced the doxorubicin-resistant phenotype of MCF-7 and BT474 cells. But treatment with fulvestrant obviously reversed WBP2 upregulation mediated the increase of chemoresistance, which suggested that ER inhibitor blunted WBP2-induced chemoresistance characteristics in ER-positive breast cancer cells (Fig. 6e). However, it will need many more studies to overcome WBP2-mediated drug resistance before combination of ER inhibitor (such as fulvestrant) and chemotherapy can be used for clinical practice. Collectively, we confirmed that WBP2 upregulation promoted the interaction of WBP2 and ERα, followed by the enhanced transcriptional activity of ERα, which subsequently directly bounded to the promoter region of MDR1 to modulate *MDR1* transcription and affected the doxorubicin resistance of MCF-7 cells (Fig. 6f).

## Discussion

A critical challenge for chemotherapy is mainly from the gradual induction of chemoresistance in breast cancer. However, the underlying molecular mechanisms are not yet clear. The data presented here indicates that WBP2 contributes to chemoresistance in ERα-positive breast cancer cells, as shown in an in vivo animal model and in vitro cell experiments.

WBP2, a binding partner of WW domain proteins, mediates the signalling pathway of intracellular downstream molecules that possess the WW domain or SH3 domain, including WWOX,^23^ Nedd4,^30^ Rsp5p^31^ and TAZ.^20^ In mammals, WBP2 interacts biochemically with Pax8 and is required for morphogenesis of the thyroid gland.^32^ Emerging research indicates that WBP2 acts as an oncogene in glioma by affecting cancer cell metabolism.^33^ In fact, most studies about WBP2 are mainly focused on its role in breast cancer. Upregulation of WBP2 expression or its tyrosine phosphorylation level in breast cancer cells promotes tumour cell growth and metastasis via modulating ER,^22^ Hippo^34^ and Wnt signalling networks.^24^ Based on the correlation between WBP2 expression and breast cancer, we performed genome-wide mRNA screen using WBP2 stable expressed MCF-7 cells. Combining of the differentially expressed genes observed from RNA sequencing (Fig. S5A-B), KEGG pathway enrichment analysis was carried. We found that WBP2 upregulation induced several signalling pathway changes in MCF-7 cells, such as systemic lupus erythematosus, alcoholism, viral carcinogenesis, necroptosis and tight junction (Top5) (Table S1), implying that as a co-activator, WBP2 overexpression could induce genome-wide changes and affect several signalling transduction in breast cancer cells. In this study, we also evaluated WBP2 expression in ERα-positive drug-resistant MCF-7/ADR and ERα-negative drug-resistant MDA-MB-231/ADR cell lines. Our data illustrated that WBP2 was overexpressed only in ERα-positive MCF-7/ADR cells compared with MCF-7 cells, but not in ERα-negative drug-resistant cell lines. These findings linked WBP2 and chemoresistance together for the first time and enriched the understanding of the function of WBP2 in tumour cells.

An active efflux mechanism is the main reason for multidrug resistance (MDR) of tumour cells to chemotherapeutic agents. MDR1 is an important component of ATP-dependent efflux pumps that pump many foreign substances out of cells.^35^ A variety of chemotherapeutic agents including doxorubicin, paclitaxel and cisplatin are known to be the substrates of P-glycoprotein.^36, 37^ Overexpression of MDR1 could be conducive to both initial and induced chemotherapy resistance of breast cancer cells, resulting in increased resistance of cancer cells to medications.^38, 39^ Here, through cell viability and apoptosis assays, we revealed that WBP2 inhibition enhanced the sensitivity of ERα-positive drug-resistant MCF-7/ADR cells to chemotherapy agents through upregulation of MDR1 expression. However, we did not observe any changes of cell viability after treatment with doxorubicin in ERα-negative breast cancer cells. Our findings provide a novel insight into how ERα signalling regulates the response of ERα-positive breast tumours to chemotherapy, and to the best of our knowledge, is the first evidence of WBP2-mediated drug resistance in ERα-positive breast cancer cells.

ERα, as a nuclear transcriptional factor, regulates target genes either through directly binding to the specific oestrogen response element (ERE) of promoters or by binding to the promoter by interacting with other transcriptional factors via an ERE-independent mechanism, such as Sp1, c-Fos/c-JunB and NF-kappa B.^19, 40–42^ Previous reports indicated that the *MDR1* promoter domain possesses inverted CCAAT BOX and GC-rich elements that can interact with Sp1, and a half-ERE sequence that can directly bind to ERα.^43–45^ To sum up, ERα may affect *MDR1* transcription through these two channels. In the present study, we posit that ERα interacts with WBP2 to enhance the transcriptional level of *MDR1*, resulting in chemoresistance of breast cancer cells by increasing membrane drug pump function. Combination of ER inhibitor fulvestrant and doxorubicin obviously reversed WBP2 upregulation mediated the increase of chemoresistance in ER-positive breast cancer cells. Possibly, both ablation of WBP2 in drug-resistant cancer cells and combination of ER inhibitor and chemotherapy facilitated the absorption of chemotherapy drugs and sensitised ERα-positive drug-resistant cancer cells to chemotherapeutics. Previous study has indicated that E3 ligase ITCH can bind and target WBP2 for ubiquitin-dependent proteasomal degradation resulting in the downregulation of WBP2 level and dysfunction in cancer. Conversely, ITCH silencing can elevate WBP2 levels.^25^ Possibly, ITCH mutant or reduction blocks the proteasomal degradation pathway acting on WBP2 expression in MCF-7/ADR cells, implying targeting ITCH or proteasomal degradation pathway might be also used for overcoming WBP2 overexpression evoked resistance to chemotherapy.

In conclusion, this study demonstrated that WBP2 directly promoted P-glycoprotein expression through binding to ERα to increase the doxorubicin resistance of ERα-positive MCF-7 cells via systematic in vitro and in vivo investigations. Our findings provide a novel mechanism of ERα-mediated chemoresistance and suggest that WBP2 might be an effective target for the development of novel therapeutic strategies to overcome multiple drug resistance in breast cancer.

## Electronic supplementary material

Supplementary Figures

Supplementary Table 1
