# Supplementary material for: Interaction of WBP2 with ERα increases doxorubicin resistance of breast cancer cells by modulating MDR1 transcription
Source: Br J Cancer. 2018 Jun 25;119(2):182–92. doi: 10.1038/s41416-018-0119-5 (PMC6048156; doi:10.1038/s41416-018-0119-5)
Supplement: Supplementary file 1 — Supplementary Figures [file 41416_2018_119_MOESM1_ESM.pdf]

Fig. S1

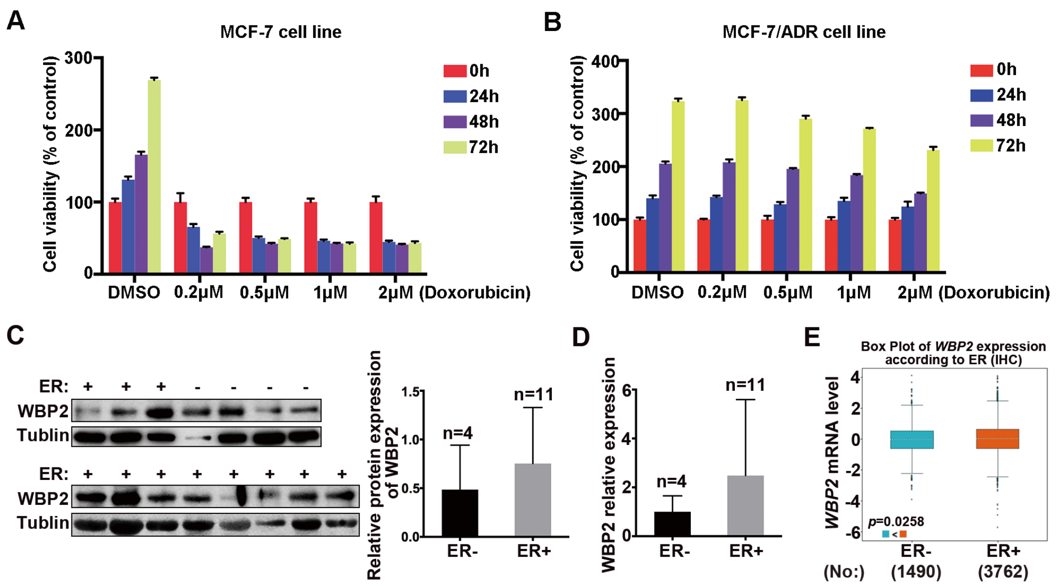

Fig. S1. Validation of doxorubicin resistant MCF-7/ADR cells and evaluation of WBP2 expression in ER positive and ER negative patients. (A) and **(B)** Cell viabilities of MCF-7 and MCF-7/ADR cells after treatment with Doxorubicin. **(C)** and **(D)** Protein and mRNA levels of WBP2 in ER positive and ER negative breast cancer patients (ER+: n=11, ER-: n=4). **(E)** Differential expression of WBP2 mRNA between 1490 ER negative and 3762 ER positive breast cancer patients.

Fig. S2

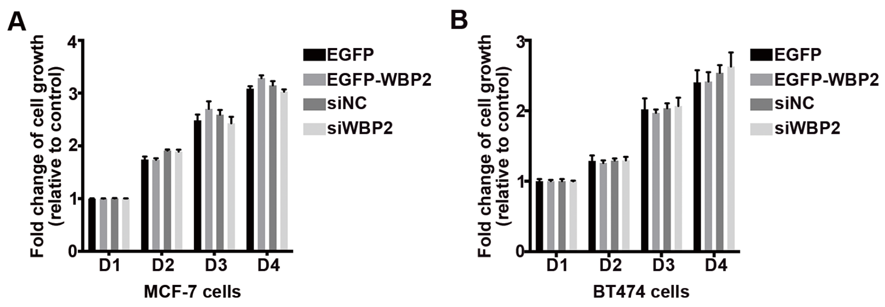

Fig. S2. Effects of WBP2 on growth ability in ER positive cells. (A) Cell viabilities of WBP2 overexpressed/silenced MCF-7 cells. **(B)** Cell viabilities of WBP2 overexpressed/silenced BT474 cells.

Fig. S3

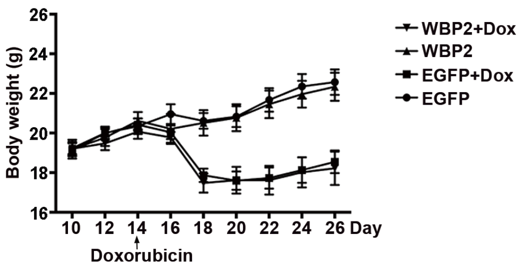

Fig. S3. Body weight of four group breast cancer bearing mice. WBP2+Dox: WBP2 overexpressed MCF-7 cells treated with doxorubicin. WBP2: WBP2 overexpressed MCF-7 cells. EGFP+Dox: Control MCF-7 cells treated with doxorubicin. EGFP: Control MCF-7 cells.

Fig. S4

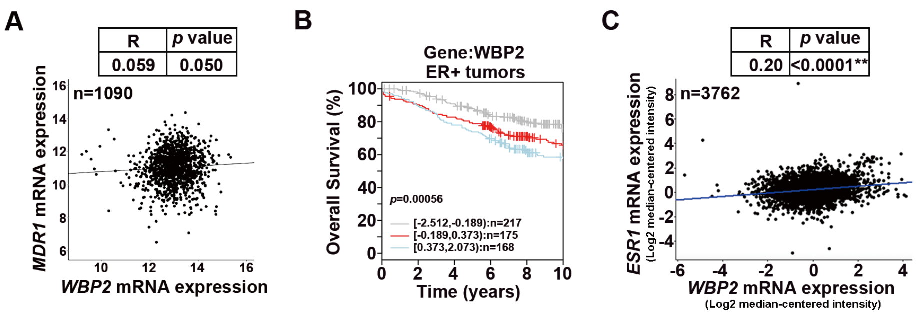

Fig. S4. Correlation analysis among WBP2, MDR1, and ESR1 using online available dataset. (A) The correlation analysis between MDR1 and WBP2 using TCGA dataset. **(B)** The correlation analysis between WBP2 expression and progression and poor clinical outcomes of ER positive tumors utilizing GOBO Gene Set Analysis. N indicates samples number. –[-2.512,-0.189), –[-0.1890.373), –[0.3732.073) indicate Log2 median-centered intensity of *WBP2* mRNA in all these samples. **(C)** The correlation analysis between WBP2 and ER (ESR1) in ER positive breast cancer patients by using Breast Cancer Gene-Expression Miner v4.1. R indicates Pearson's coefficient.

Fig. S5

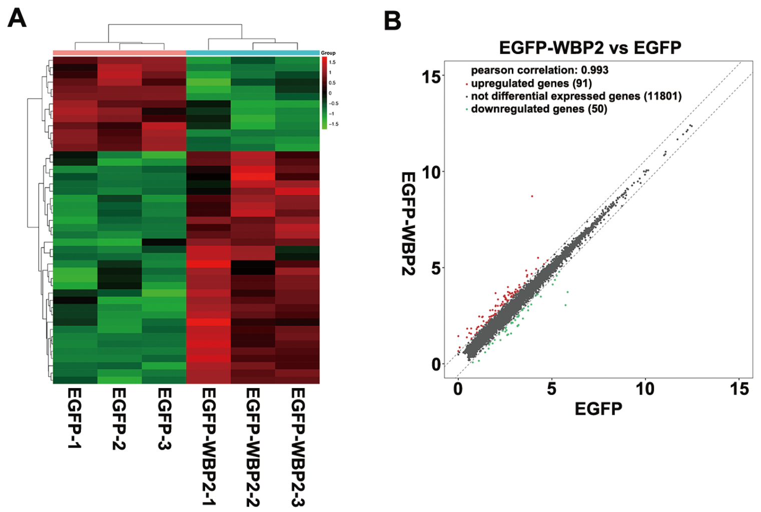

Fig. S5. Results of genome wide mRNA screen assay in MCF-7 cells. (A) Heat map of differentially expressed genes between EGFP and EGFP-WBP2 cells. **(B)** Scatter plot of differentially expressed genes between EGFP and EGFP-WBP2 cells. EGFP-WBP2: WBP2 overexpressed MCF-7 cells. EGFP: Control MCF-7 cells.
