# Supplementary material for: Interaction of WBP2 with ERα increases doxorubicin resistance of breast cancer cells by modulating MDR1 transcription
Source: Br J Cancer. 2018 Jun 25;119(2):182–92. doi: 10.1038/s41416-018-0119-5 (PMC6048156; doi:10.1038/s41416-018-0119-5)
Supplement: Supplementary file 2 — Supplementary Table 1 [file 41416_2018_119_MOESM2_ESM.docx]

| **ID** | **Term** | **Count** | ***p* value** | **FDR** |
| --- | --- | --- | --- | --- |
| **Up Differentially Expressed Genes KEGG pathway** | | | | |
| Hsa05322 | Systemic lupus erythematosus | 6 | 1.48e-07 | 9.03e-06 |
| Hsa05034 | Alcoholism | 6 | 8.91e-07 | 2.72e-05 |
| Hsa05203 | Viral carcinogenesis | 4 | 6.13e-04 | 1.25e-02 |
| Hsa04217 | Necroptosis | 2 | 4.40e-02 | 3.42e-01 |
| Hsa04530 | Tight junction | 2 | 4.69e-02 | 3.42e-01 |
| **Down Differentially Expressed Genes KEGG pathway** | | | | |
| Hsa05142 | Chagas disease | 2 | 1.90e-03 | 5.90e-02 |

**Table S1. KEGG pathway analysis results of differentially expressed genes (Top5)**
